# Disruption of Hepatic Insulin Signaling Causes Phospholipid Dysregulation in Mice

**DOI:** 10.1096/fj.202504306R

**Published:** 2026-02-24

**Authors:** Quan Pan, Meixia Pan, Weiqi Ai, Wanbao Yang, Wen Jiang, Xianlin Han, Shaodong Guo

**Affiliations:** ^1^ Department of Nutrition, College of Agriculture and Life Sciences Texas A&M University College Station Texas USA; ^2^ Barshop Institute for Longevity and Aging Studies University of Texas Health Science Center at San Antonio Texas USA; ^3^ Department of Medicine, Division of Diabetes University of Texas Health Science Center at San Antonio San Antonio Texas USA

**Keywords:** foxo1, insulin signaling, liver, phospholipids, TGF‐β1

## Abstract

Phospholipids are important components of the bilayer of biological membranes. Alterations of phospholipids are associated with metabolic disorders, including insulin resistance. However, how impaired insulin signaling impacts phospholipids has not been well established. Disruption of hepatic insulin signaling is achieved by insulin receptor substrate 1 (IRS1) and IRS2 double deletion (DKO) in the liver. Further deletion of TGF‐β1 or Foxo1 in the liver of DKO mice was used to examine the role of TGF‐β1 or Foxo1 in contributing to the alterations of phospholipid metabolism in DKO mice. Disruption of hepatic insulin signaling led to the dysregulation of phospholipids, including phosphatidylcholine (PC), phosphatidylethanolamine (PE), phosphatidylinositol (PI), phosphatidylserine (PS), sphingomyelin (SM), cardiolipin (CL), and lysophospholipids in the liver. Mechanistically, disruption of hepatic insulin signaling dysregulated the expression of genes related to phospholipid metabolism. Interestingly, further deletion of *Tgfb1* in the liver of DKO mice (TKObeta1) attenuated the alterations of phospholipids and rescued the abnormal expression of genes related to phospholipid metabolism. Moreover, deletion of transcription factor *Foxo1*, a key mediator of insulin signaling, achieved similar beneficial effects as *Tgfb1* deletion in DKO mice. Our study suggests that insulin signaling plays a crucial role in maintaining phospholipids balance in the liver via TGF‐β1 or Foxo1. Targeting TGF‐β1 or Foxo1 could be promising strategies to combat phospholipids alterations and related metabolic dysfunctions.

## Graphical abstract

*Pan et al.* demonstrate that disruption of hepatic insulin signaling by insulin receptor (IR) substrate (IRS1/2) deletion led to impaired PI3K‐AKT signaling, causing increased Foxo1 activation and TGF‐β1 expression, which dysregulated the expression of the key enzymes involving phospholipid biosynthesis and remodeling. This research reveals the crucial role of Insulin signaling in maintaining phospholipids balance in the liver via TGF‐β1 or Foxo1.

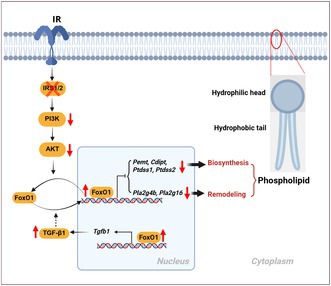

**Abbreviations**
CDP‐choline: (Cytidine diphosphate‐choline)
CDP‐DAG: (Cytidine diphosphate‐diacylglycerol)
CDP‐Etn: (Cytidine diphosphate‐ethanolamine)
Chpt: (CDP‐choline:1,2‐diacylglycerol cholinephosphotransferase)
CL: (Cardiolipin)
CNTR: (Control)
CPT: (Cytidine diphosphate‐choline:1,2‐diacylglycerol cholinephosphotransferase)
DAG: (Diacylglycerol)
DKO: (Double knockout—liver‐specific Irs1 and Irs2 knockout)
EPT: (Ethanolamine phosphotransferase)
Foxo1: (Forkhead box protein O1)
Irs1: (Insulin receptor substrate 1)
Irs2: (Insulin receptor substrate 2)
LCL: (Lysocardiolipin)
LPC: (Lysophosphatidylcholine)
LPCAT: (Lysophosphatidylcholine acyltransferase)
LPE: (Lysophosphatidylethanolamine)
MLCLAT: (Monolysocardiolipin acyltransferase)
NAFLD: (Nonalcoholic fatty liver disease)
PA: (Phosphatidic acid)
PC: (Phosphatidylcholine)
PE: (Phosphatidylethanolamine)
PEMT: (Phosphatidylethanolamine methyltransferase)
PG: (Phosphatidylglycerol)
PI: (Phosphatidylinositol)
PIS: (Phosphatidylinositol synthase)
PLA2: (Phospholipase A2)
PLA2g16: (Phospholipase A2 group XVI)
PLA2g4b: (Phospholipase A2 group IVB)
Pnpla6: (Patatin‐like phospholipase domain‐containing 6)
Pnpla7: (Patatin‐like phospholipase domain‐containing 7)
PS: (Phosphatidylserine)
PSD: (Phosphatidylserine decarboxylase)
PSS1: (Phosphatidylserine synthase 1)
PSS2: (Phosphatidylserine synthase 2)
Sgms: (Sphingomyelin synthase)
SM: (Sphingomyelin)
SMS: (Sphingomyelin synthase)
TGF‐β1: (Transforming growth factor beta 1)
TKObeta1: (Triple knockout for Irs1, Irs2, and Tgfb1)
TKOfoxo1: (Triple knockout for Irs1, Irs2, and Foxo1)
VLDL: (Very low‐density lipoproteins)

## 1 Introduction

Eukaryotic cells contain three main lipid classes in plasma membrane and intracellular organelle membranes: glycerophospholipids containing a glycerol backbone, sphingolipids containing a sphingosine backbone, and sterols containing a four‐ringed structure [1]. Glycerophospholipids mainly include phosphatidylcholine (PC), phosphatidylethanolamine (PE), phosphatidylinositol (PI), phosphatidylglycerol (PG), phosphatidic acid (PA), cardiolipin (CL), and phosphatidylserine (PS) [2]. The major sphingolipids in mammalian non‐nerve cells are sphingomyelins (SM). Catabolism of glycerophospholipids and sphingolipids produces messenger lipids: lysoPC (LPC), lysoPE (LPE), lysoCL (LCL), diacylglycerol (DAG), ceramide, etc. These phospholipids are important in maintaining plasma and organelle membrane integrity, homeostasis, and function, and participate in cellular signaling transductions [3]. Altered phospholipid metabolism is associated with the pathogenesis of various metabolic diseases, including obesity, diabetes, aging, and nonalcoholic fatty liver disease (NAFLD) [4, 5, 6].

Insulin is a pancreatic beta cells‐secreted hormone, targeting multiple organs or tissues including the liver, adipose tissue, and muscle to regulate glucose, protein and lipid metabolism [7]. In the liver, insulin stimulates glucose uptake, promotes glycogen, protein and lipid synthesis. In addition, hepatic insulin signaling inhibits gluconeogenesis via suppressing the activity of transcription factor forkhead box protein O1 (Foxo1) and its target gluconeogenic genes expression. Hepatic insulin resistance, a pathophysiological state where insulin action is impaired the liver, is associated with metabolic syndrome and NAFLD [8]. Previous studies have shown that altered phospholipid metabolism is associated with insulin resistance development [5, 9]. However, these studies majorly focus on how phospholipid alterations affect the pathological process of insulin resistance [5, 10]. The effects of impaired insulin signaling on phospholipid profiles have not been well established.

In this study, we aimed to examine the role of insulin signaling in control of phospholipid metabolism. With our unique hepatic insulin signaling disruption mouse model (DKO), we determined the effects of hepatic insulin signaling disruption on phospholipid profiling in the liver. Lipidomic analysis demonstrated profound alterations of phospholipids in the liver of DKO mice. Moreover, deletion of insulin downstream targets: transforming growth factor beta 1 (TGF‐β1) or Foxo1 could attenuate such alterations in DKO mice. Targeting TGF‐β1 or Foxo1 could be promising strategies to combat phospholipid alterations and ‐associated metabolic diseases.

## 2 Research Design and Methods

### 2.1 Animals

Liver‐specific *Irs1* and *Irs2* double knock‐out (DKO) mice, liver‐specific *Irs1*, *Irs2* and *Tgfb1* triple knock‐out (TKObeta1) mice, and liver‐specific *Irs1*, *Irs2* and *Foxo1* triple knock‐out (TKOfoxo1) mice were generated as previously described [11]. *Irs1*, *Irs2* floxed mice served as the control (CNTR) for DKO mice, and *Irs1*, *Irs2*, *Tgfb1* floxed mice served as the control (CNTR3) for TKObeta1 mice. All mice used in this study were male, maintained at 22°C in a 12/12 h light–dark cycle, and given free access to food and water. All animal experiments were performed according to procedures approved by Texas A&M University Institutional Animal Care and Use Committee.

### 2.2 Lipid Extraction

Mouse livers were collected after PBS perfusion under *ad libitum* feeding conditions. Liver tissues were homogenized in ice‐cold 10‐times diluted PBS. Lipids were extracted by a modified procedure of Bligh and Dyer extraction as previously described [12, 13]. Briefly, internal standard mixtures were added to samples based on total protein content of each individual sample. 12 mL of chloroform/methanol/50 mM lithium chloride solution (1:1:1) were added to samples and vortexed for 1 min, then centrifuged at 4000 × g for 10 min. The chloroform layer was collected. An additional 4 mL of chloroform was added to the methanol/aqueous layer. After vortexing and centrifugation, the chloroform layer was collected and combined with the first extract and dried under a nitrogen stream. This extraction procedure was repeated once by adding 12 mL of chloroform/methanol/10 mM lithium chloride solution (1:1:1) to the lipid residues. The chloroform layer was collected and dried under a nitrogen stream. Finally, each lipid extract was resuspended with a volume of 500 μL of chloroform/methanol (1:1) per mg of original tissue protein. Individual lipid extracts were further diluted with chloroform/methanol/isopropanol (1:2:4) prior to direct infusion with a NanoMate device for MS analysis.

### 2.3 Mass Spectrometric Analysis

Mass spectrometry (MS) and tandem MS analyses of lipids were performed as previously described [14, 15]. Tandem MS analysis of a diluted lipid solution was conducted under fixed collision gas pressure of 1.0 mTorr [14]. All mass spectral data were automatically acquired using a customized sequence subroutine operated under Xcalibur software [15]. Data processing was conducted as previously described [14] after considering the principles of shotgun lipidomics [16, 17].

### 2.4 RNA Sequencing

The liver samples were sent to the Texas A&M Institute for Genome Sciences and Society (TIGSS) molecular genomics laboratory for RNA extraction and RNA sequencing. RNA sequencing was performed on the NovaSeq 6000 (Illumina, San Diego, CA, USA) instrument that generated 150‐base‐pair, paired‐end sequences. The sequencing run produced approximately 6 million reads per sample and resulted in ~200 X coverage for each sample.

### 2.5 Metabolomic Analysis

Mouse livers were collected after PBS perfusion. Liver samples were sent to Metabolon Inc. (Durham, NC, USA) for metabolomic analysis. Data processing was conducted using a custom programmed Microsoft Excel macro.

### 2.6 Statistics

Data are generally presented as mean ± SEM. Student's two‐tailed *t*‐tests were used when comparing the differences between two groups, and one‐way ANOVA tests followed by Tukey's multiple comparisons tests were used when comparing two groups among multiple groups; *p* < 0.05 was considered statistically significant.

## 3 Results

### 3.1 Disruption of Hepatic Insulin Signaling Causes Dysregulation of Phosphatidylcholine (PC) Profiles in the Liver

**FIGURE 1:**
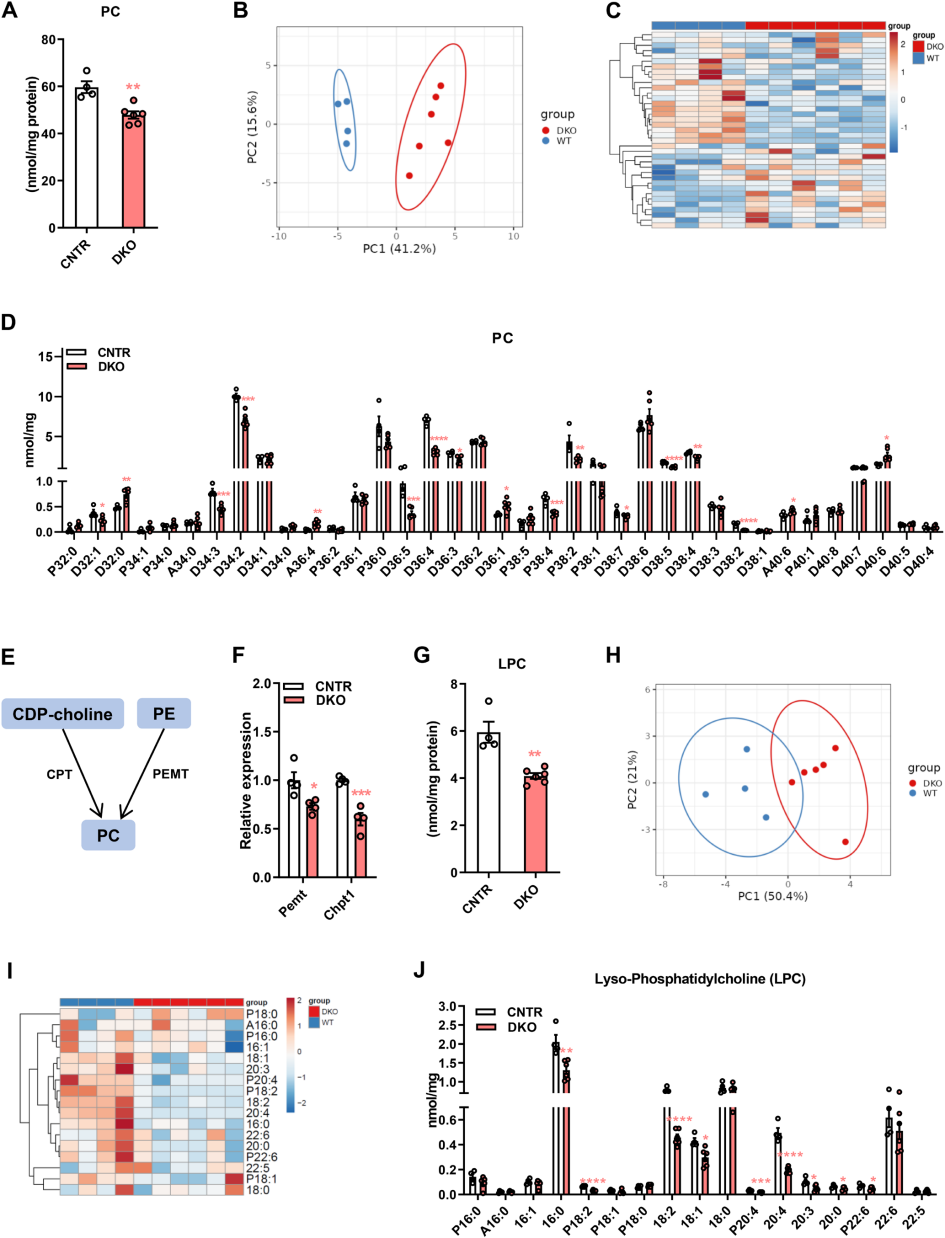
Disruption of hepatic insulin signaling causes remodeling of phosphatidylcholine (PC) profiles in the liver. (A) Total PC levels in the liver of DKO and control (CNTR) mice. (B and C) Principal component analysis (B) and heatmap (C) of haptic PC molecular species in DKO and WT mice. (D) Levels of haptic PC molecular species in DKO and CNTR mice. (E) Diagram showing PC synthesis: CDP‐choline is converted to PC by CPT; PE is converted to PC by PEMT. (F) Relative expression of *Pemt* and *Chpt1* in the liver of DKO and CNTR mice. (G) LPC levels in the liver of DKO and CNTR mice. (H and I) Principal component analysis (H) and heatmap (I) of haptic LPC molecular species in DKO and WT mice. (J) Levels of haptic LPC molecular species in DKO and CNTR mice. PC, phosphatidylcholine; DAG, diacylglycerol; CPT, cytidine diphosphate‐choline:1,2‐diacylglycerol cholinephosphotransferase; PE, phosphatidylethanol‐amine; PEMT, phosphatidylethanolamine methyltransferase; LPC, lyso‐phosphatidylcholine. Data are presented as the means ± SEM. *p < 0.05, **p < 0.01, **p < 0.001, ****p < 0.0001 vs. CNTR group using *t*‐test.

PC is the most abundant phospholipid in cells [4]. To determine the effect of impaired insulin signaling on PC profiles, we disrupted hepatic insulin signaling by deletion of *Irs1* and *Irs2* in the liver (DKO) and found that DKO mice had significantly lower total hepatic PC levels (Figure 1A). Principal component analysis demonstrated that hepatic PC profiles of DKO mice were clearly separated from those of CNTR mice (Figure 1B). Several PC species were altered in the liver of DKO mice, including diacyl (D)32: 1, D32: 0, D34: 3, D34: 2, alkyl ether (A)36: 4, D36: 5, D36: 4, D36: 1, plasmalogen (P)38: 4, P38: 2, D38: 7, D38: 5, D38: 4, A40: 6, and D40: 6 (Figure 1C,D). In mammalian cells, PC is synthesized from cytidine diphosphate‐choline (CDP‐choline) via the Kennedy pathway, also called the CDP‐choline pathway, which is regulated by CDP‐choline:1,2‐diacylglycerol cholinephosphotransferase (CPT, encoded by the *Chpt* gene) (Figure 1E). Additionally, hepatocytes have a unique phosphatidylethanolamine (PE) methyltransferase (PEMT, encoded by the *Pemt* gene) for PC synthesis from PE via the PEMT pathway (Figure 1E) [18]. Interestingly, disruption of hepatic insulin signaling notably reduced *Chpt1* and *Pemt* expression in the liver (Figure 1F), contributing to the decrease of total PC levels in DKO mice. PC can be hydrolyzed by phospholipase A2 (PLA2) to generate lyso‐choline (LPC), which regulates lipid metabolism in hepatocytes [19]. We found that disruption of insulin signaling significantly decreased LPC levels in the liver (Figure 1G). Although the hepatic LPC profile of DKO was not clearly separated from those of CNTR mice, several LPC species were significantly lower in DKO mice, including 16: 0, P18: 2, 18: 2, 18: 1, 20: 4, 20: 3, 20: 0, and P22: 6 (Figure 1H–J). These results demonstrate that disruption of insulin signaling reshapes PC and LPC profiles in the liver.

### 3.2 Disruption of Hepatic Insulin Signaling Causes Dysregulation of Phosphatidylethanolamine (PE) Profiles in the Liver

**FIGURE 2:**
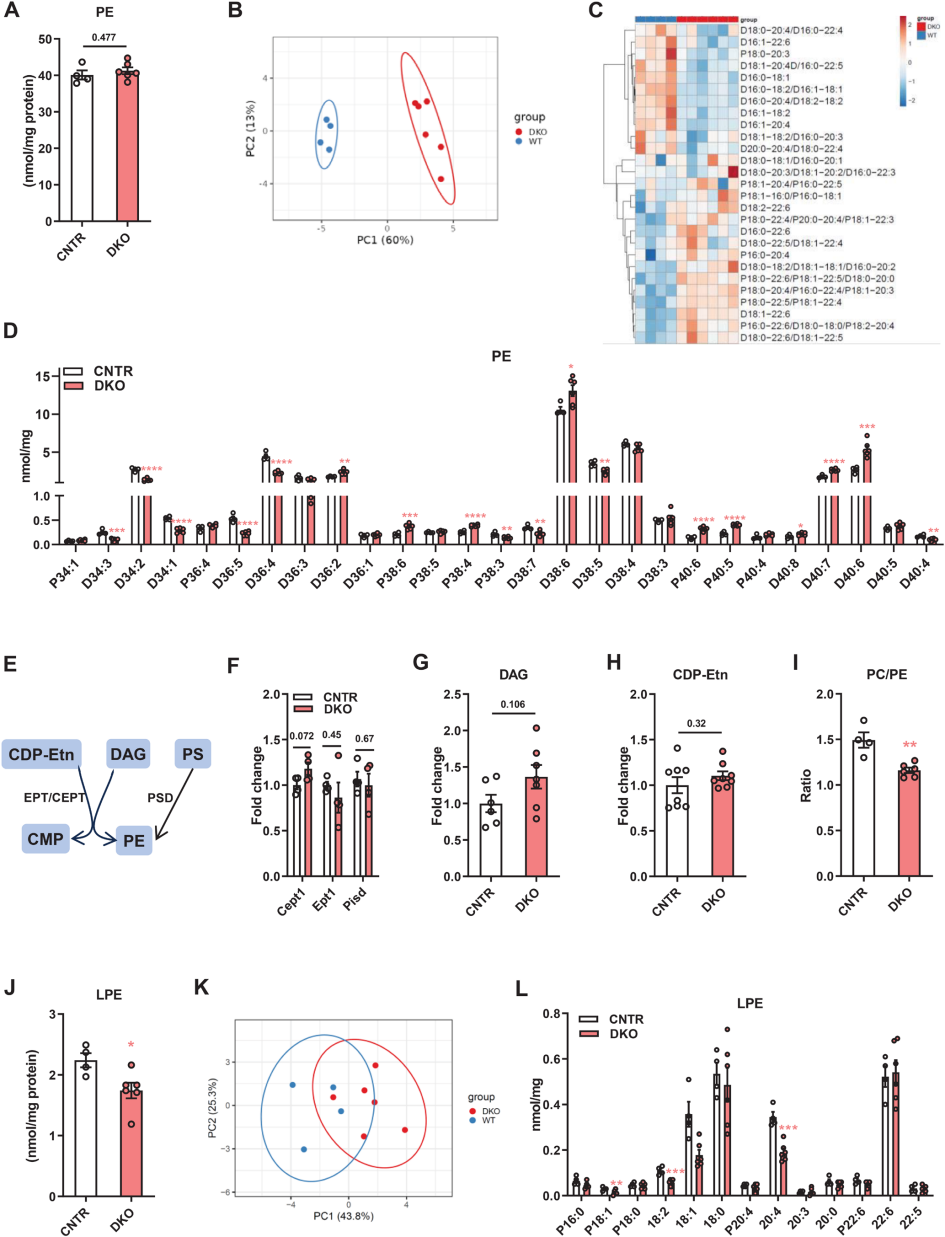
Disruption of hepatic insulin signaling causes remodeling of phosphatidylethanolamine (PE) profiles in the liver. (A) Total PE levels in the liver of DKO and CNTR mice. (B and C) Principal component analysis (B) and heatmap (C) of hepatic PE molecular species in DKO and WT mice. (D) Levels of hepatic PE molecular species in DKO and CNTR mice. (E) Diagram showing PE synthesis: DAG and CDP‐Etn are converted to PC by EPT/CEPT; PS is converted to PE by PSD. (F) Relative expression of *Cepts, Ept1* and *Pisd* in the liver of DKO and CNTR mice. (G and H) Hepatic DAG (G) and CDP‐Etn (H) levels in DKO and CNTR mice. (I) Hepatic PC to PE ratio in DKO and CNTR mice. (J) LPC levels in the liver of DKO and CNTR mice. (K) Principal component analysis of hepatic LPE molecular species in DKO and WT mice. (L) Levels of hepatic LPE molecular species in DKO and CNTR mice. CDP‐Etn, cytidine diphosphate‐ethanolamine; DAG, diacylglycerol; PE, phosphatidylethanolamine; CMP, cytidine monophosphate; PS, phosphatidylserine; CEPT, choline/ethanolamine phosphotransferase; EPT, ethanolamine phosphotransferase; PSD, phosphatidylserine decarboxylase; LPE, lyso‐phosphatidylethanol‐amine. Data are presented as the means ± SEM. *p < 0.05, **p < 0.01, **p < 0.001, ****p < 0.0001 vs. CNTR group using t‐test.

PE is the second most abundant phospholipid in mammalian cells [20]. Although no significant difference in total hepatic PE levels was observed between DKO and CNTR mice, hepatic PE profiles of DKO mice were clearly separated from those of CNTR mice (Figure 2A,B). Several PE species, including D34:3, D34:2, D34:1, D36:5, D36:4, D36:2, P38:6, P38:4, P38:3, D38:7, D38:6, D38:5, P40:6, P40:5, P40:8, D40:7, D40:6, and D40:4, were altered in the liver of DKO mice (Figure 2C,D). PE is synthesized from CDP‐ethanolamine (CDP‐Etn) and DAG by choline/ethanolamine phosphotransferase (CEPT, encoded by *Cept* gene) and ethanolamine phosphotransferase (EPT, encoded by *Ept* gene) or from PS by phosphatidylserine decarboxylase (PSD, encoded by *Pisd* gene) (Figure 2E) [4]. Disruption of hepatic insulin signaling had no significant impact on *Cept1*, *Ept1*, and *Pisd* expression, and hepatic DAG and CDP‐Etn levels, which explains the comparable total PE levels observed in the liver of DKO and CNTR mice (Figure 2F–H). However, the PC/PE ratio, which is crucial to maintain membrane integrity and permeability [2], was notably decreased in DKO mice (Figure 2I). PE can be hydrolyzed by PLA1/PLA2 to generate lyso‐PE (LPE) [21]. Here, we found that disruption of insulin signaling significantly decreased LPE levels in the liver (Figure 2J). Although there is no clear separation of hepatic LPE profiles between DKO and CNTR mice, several hepatic LPE species, including P18:1, 18:2, and 20:4, were significantly lower in DKO mice (Figure 2K,L). These results demonstrate that disruption of insulin signaling leads to the remodeling of PE and LPE profiles in the liver.

### 3.3 Disruption of Hepatic Insulin Signaling Causes Dysregulation of Phosphatidylinositol (PI) Profiles in the Liver

**FIGURE 3:**
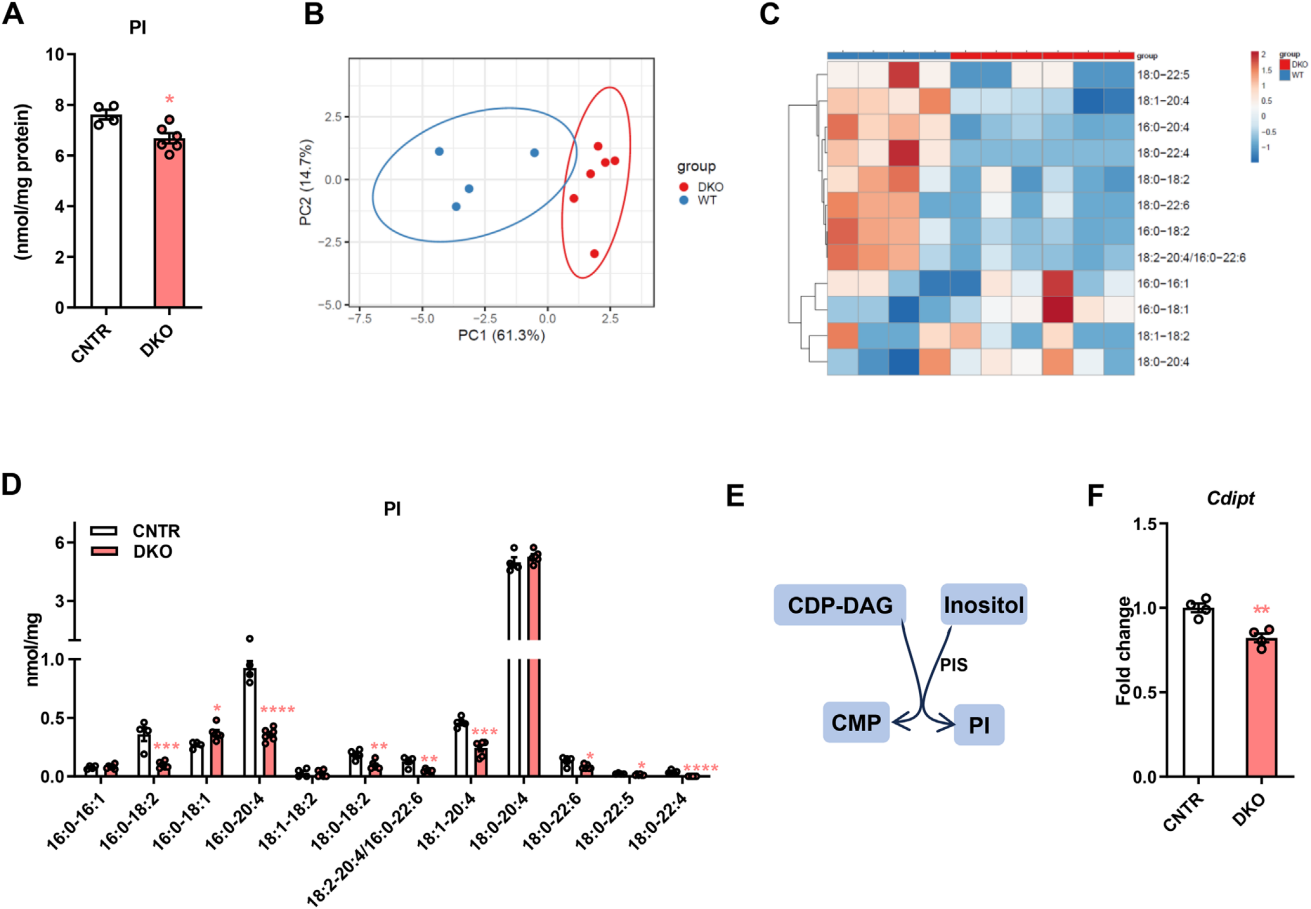
Disruption of hepatic insulin signaling causes remodeling of phosphatidylinositol (PI) profiles in the liver. (A) Total PI levels in the liver of DKO and CNTR mice. (B and C) Principal component analysis (B) and heatmap (C) of hepatic PI molecular species in DKO and WT mice. (D) Levels of hepatic PI molecular species in DKO and CNTR mice. (E) Diagram showing PI synthesis: CDP‐DAG and inositol are converted to PC by EPT/CEPT; inositol is converted to PI by PIS. (F) Relative expression of *Cdipt* in the liver of DKO and CNTR mice. CDP‐DAG, cytidine diphosphate diacylglycerol; CMP, cytidine monophosphate; PI, phosphatidylinositol; PIS, phosphatidylinositol synthase. Data are presented as the means ± SEM. *p < 0.05, **p < 0.01, **p < 0.001, ****p < 0.0001 vs. CNTR group using *t*‐test.

PI constitutes 5%–10% of total cellular lipids in mammalian cells and is the source for generating phosphorylated derivatives of PI [22]. We next examined the hepatic PI profiles in CNTR and DKO mice and observed a significant decrease of total PI levels in the liver DKO mice (Figure 3A). Hepatic PI profiles of DKO mice were separated from those of CNTR mice (Figure 3B). Several PI species, including 16:0–18:2, 18:0–20:4, 18:0–18:2, 18:2–20:4/16:0–22:6, 18:1–20:4, 18:0–22:6, 18:0–22:5, and 18:0–22:4, were decreased in the liver of DKO mice (Figure 3C,D). PI is synthesized from inositol and CDP‐DAG by PI synthase (PIS, encoded by *Cdipt* gene) (Figure 3E). We found that impaired insulin action caused reduced *Cdipt* expression in the liver, which potentially contributed to the reduced hepatic PI levels in DKO mice (Figure 3F).

### 3.4 Disruption of Hepatic Insulin Signaling Dysregulates Phosphatidylserine (PS) Profiles in the Liver

**FIGURE 4:**
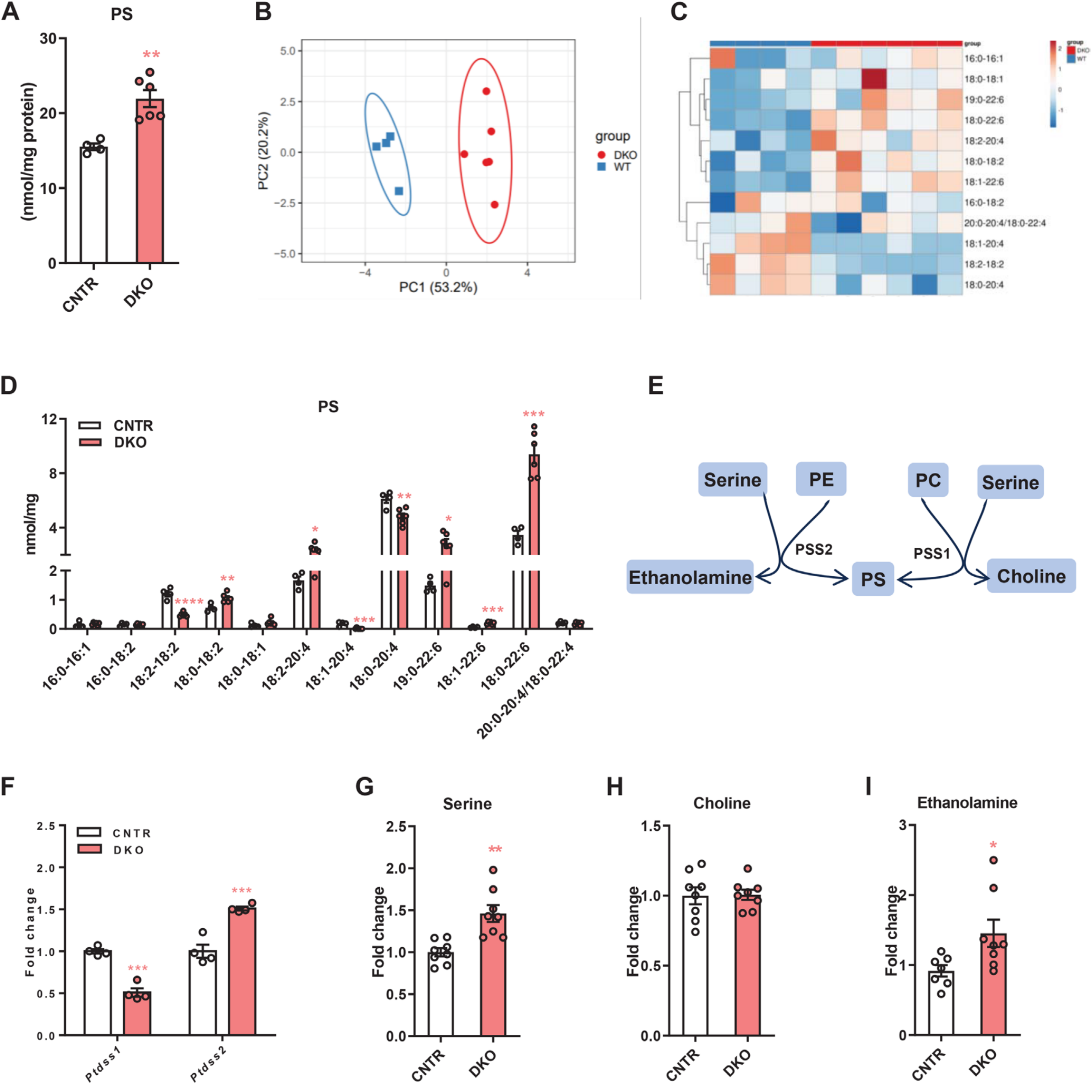
Disruption of hepatic insulin signaling causes remodeling of phosphatidylserine (PS) profiles in the liver. (A) Total PS levels in the liver of DKO and CNTR mice. (B and C) Principal component analysis (B) and heatmap (C) of haptic PS molecular species in DKO and WT mice. (D) Levels of haptic PS molecular species in DKO and CNTR mice. (E) Diagram showing PS synthesis: PE and serine are converted to PS by PSS2; PC and serine are converted to PS by PSS1. (F) Relative expression of *Ptdss1* and *Ptdss2* in the liver of DKO and CNTR mice. (G‐I) Hepatic serine (G) and choline (H), and ethanolamine (I) levels in DKO and CNTR mice. PE, phosphatidylethanolamine; PS, phosphatidylserine; PC, phosphatidylcholine; PPS, phosphatidylserine synthase. Data are presented as the means ± SEM. *p < 0.05, **p < 0.01, **p < 0.001, ****p < 0.0001 vs. CNTR group using *t*‐test.

PS is a class of the most abundant negatively charged phospholipids in mammalian cells [23]. Here, we observed a significant increase of total PS levels in the liver DKO mice (Figure 4A). Hepatic PS profiles of DKO mice were clearly separated from those of CNTR mice (Figure 4B). Several PS species were altered in the liver of DKO mice, including increased 18:0–18:2, 18:2–20:4, 19:0–22:6, 18:0–22:6 species and decreased 18:2–18:2, 18:1–20:4, 18:0–20:4 species (Figure 4C,D). PS is produced by exchanging the headgroup of PC or PE for serine in mammalian cells by PS synthase 1 (PSS1, encoded by *Ptdss1* gene) or PSS2 (encoded by *Ptdss1* gene), respectively (Figure 4E) [24]. While having lower expression of *Ptdss1*, DKO mice showed increased *Ptdss2* expression in the liver (Figure 4F). Moreover, hepatic serine levels were increased in DKO mice (Figure 4G). The elevated PS levels in the liver of DKO mice were likely a result of increased generation of PS from PE and serine. Indeed, we observed increases in ethanolamine levels but not in choline levels in the liver of DKO mice (Figure 4H,I). The decreased PS species might represent the difference resulting from the differential biosynthesis from different organelles or due to selective remodeling such as from 20:4‐containing PS molecular species to 22:6‐containing PS molecular species.

### 3.5 Disruption of Hepatic Insulin Signaling Causes Dysregulation of Sphingomyelin (SM) Profiles in the Liver

**FIGURE 5:**
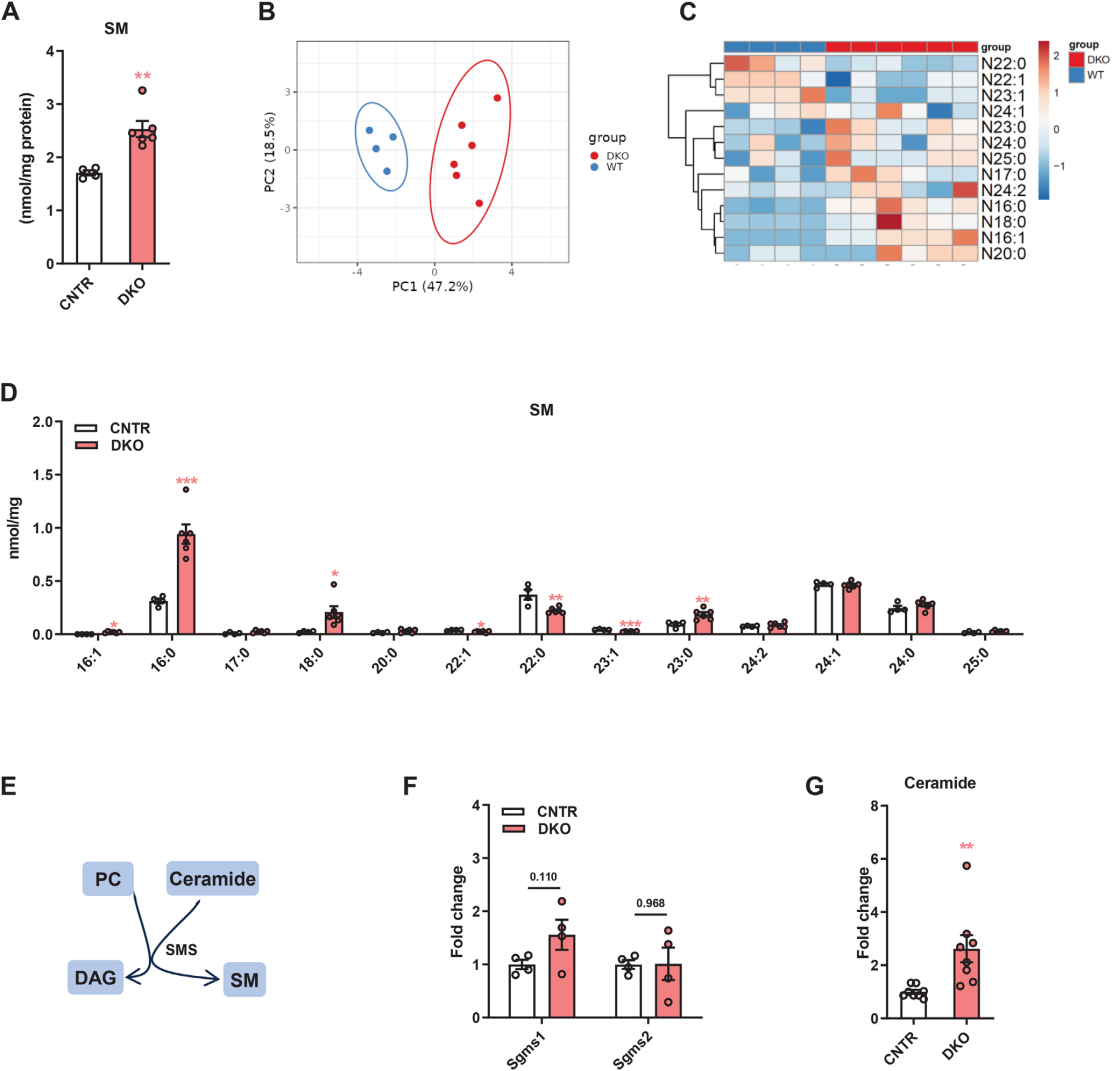
Disruption of hepatic insulin signaling causes remodeling of sphingomyelin (SM) profiles in the liver. (A) Total SM levels in the liver of DKO and CNTR mice. (B and C) Principal component analysis (B) and heatmap (C) of hepatic SM molecular species in DKO and WT mice. (D) Levels of hepatic SM molecular species in DKO and CNTR mice. (E) Diagram showing SM synthesis: PC and ceramide are converted to SM by SMS. (F) Relative expression of *Sgms1* and *Sgms2* in the liver of DKO and CNTR mice. (G) Hepatic ceramide levels in DKO and CNTR mice. SM, sphingomyelin; PC, phosphatidylcholine; DAG, diacylglycerol; SMS, sphingomyelin synthase. Data are presented as the means ± SEM. *p < 0.05, **p < 0.01, **p < 0.001, ****p < 0.0001 vs. CNTR group using *t*‐test.

SM is the most abundant sphingolipid in mammalian non‐nerve cells [3]. We found that DKO mice had higher hepatic SM levels compared to CNTR mice (Figure 5A). Hepatic SM profiles of DKO mice were clearly separated from those of CNTR mice (Figure 5B). Several SM species, especially SMs containing saturated acyl‐chains, including 16:0, 18:0, and 23:0, were elevated in the liver of DKO mice (Figure 5C,D). SM is synthesized from PC and ceramide by an enzyme named sphingomyelin synthase (SMS, encoded by the *Sgms* gene) (Figure 5E). While showing similar hepatic *Sgms1* and *Sgms2* expression as CNTR mice, DKO mice had significantly higher ceramide levels in the liver, which potentially contributed to the elevated SM levels observed in the liver of DKO (Figure 5F,G).

### 3.6 Disruption of Hepatic Insulin Signaling Causes Dysregulation of Cardiolipin (CL) Profiles in the Liver

**FIGURE 6:**
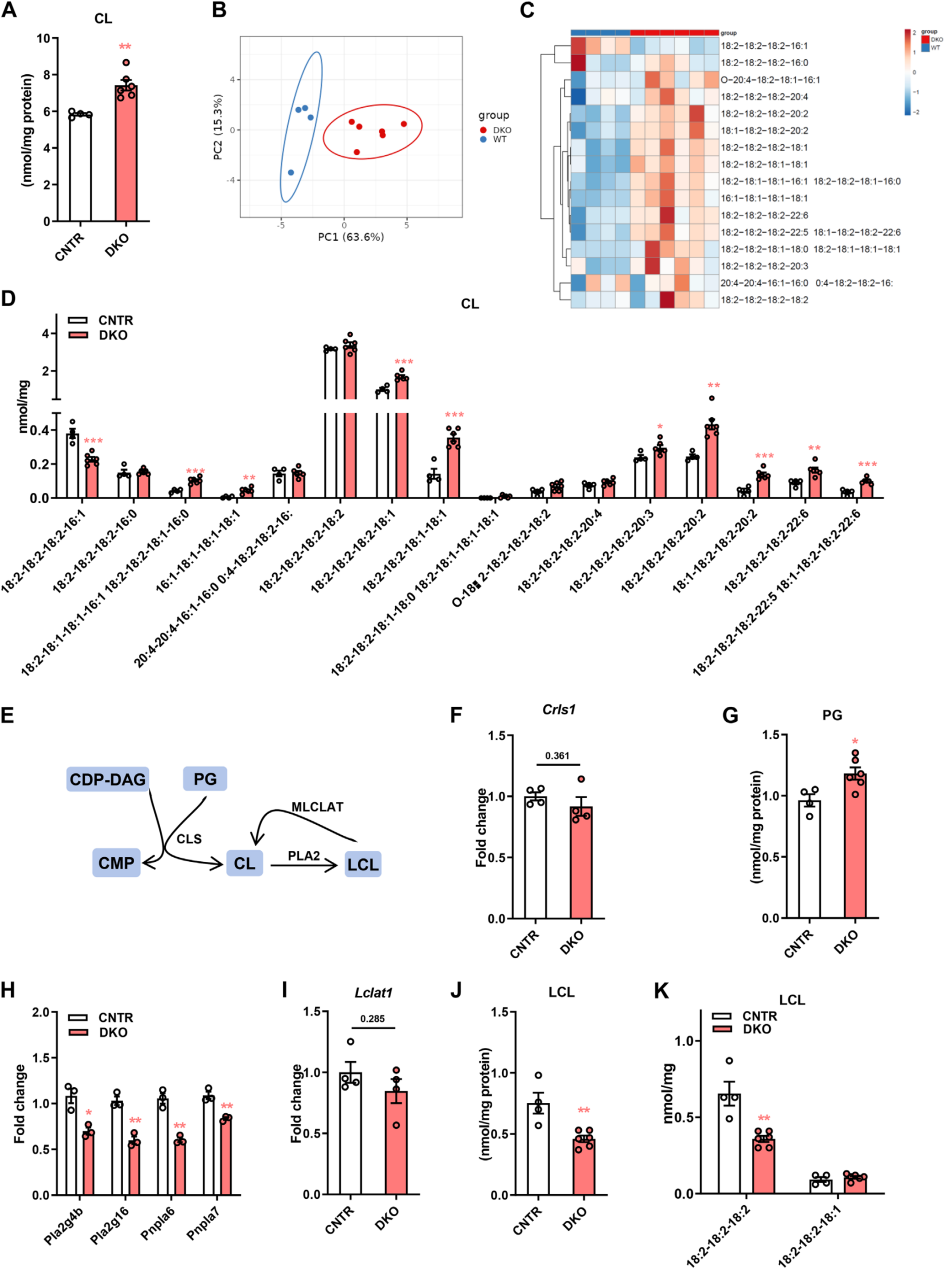
Disruption of hepatic insulin signaling causes remodeling of cardiolipin (CL) profiles in the liver. (A) Total CL levels in the liver of DKO and CNTR mice. (B and C) Principal component analysis (B) and heatmap (C) of hepatic CL molecular species in DKO and WT mice. (D) Levels of hepatic CL molecular species in DKO and CNTR mice. (E) Diagram showing CL synthesis: CDP‐DAG and PG are converted to CL by CLS; CL can be further hydrolyzed to LCL by PLA2; LCL can be converted to CL by MLCLAT. (F) Relative expression of *Crls1* in the liver of DKO and CNTR mice. (G) Hepatic PG levels in DKO and CNTR mice. (H) Relative expression of *Pla2g4b, Pla2g16, Pnpla6*, and *Pnpla7* in the liver of DKO and CNTR mice. (I) Relative expression of *Lclat1* in the liver of DKO and CNTR mice. (J) Total LCL levels in the liver of DKO and CNTR mice. (K) Levels of hepatic LCL molecular species in DKO and CNTR mice. CDP‐DAG, cytidine diphosphate diacylglycerol; PG, phosphatidylglycerol; CMP, cytidine monophosphate; CL, cardiolipin; LCL, lyso‐cardiolipin; CLS, cardiolipin synthase; PLA2, phospholipase A2; MLCLAT, monolysocardiolipin acyltransferase. Data are presented as the means ± SEM. *p < 0.05, **p < 0.01, **p < 0.001, ****p < 0.0001 vs. CNTR group using *t*‐test.

CL is a class of mitochondria exclusive phospholipids essential for mitochondrial function and dynamics [25]. We found that disruption of hepatic insulin signaling significantly led to increased CL levels in the liver (Figure 6A). Hepatic CL profiles of DKO mice were clearly separated from those of CNTR mice (Figure 6B). Several CL species, including 18:2–18:1–18:1–16:1, 18:2–18:2–18:1–16:0, 16:1–18:1–18:1–18:1, 18:2–18:2–18:1–18:1, 18:2–18:2–18:2–20:3, 18:2–18:2–18:2–20:2, 18:1–18:2–18:2–20:2, 18:2–18:2–18:2–22:6, 18:2–18:2–18:2–22:5 and 18:1–18:2–18:2–22:6, were elevated in the liver of DKO mice (Figure 6C,D). CL is synthesized from phosphatidylglycerol (PG) and CDP‐DAG by cardiolipin synthase (CLS, encoded by *Crls1* gene) (Figure 6E). While showing similar hepatic *Crls1* expression as CNTR mice, DKO mice had significantly higher PG levels in the liver, which potentially contributed to the elevated CL levels in DKO (Figure 6F,G). Besides, deaclylation of CL by PLA2 (encoded by *Pla2*) or acylation of lyso‐cardiolipin (LCL) by monolyso‐cardiolipin acyltransferase (MLCLAT, encoded by *Lclat*) also affects CL levels (Figure 6E). Intriguingly, we found that disruption of hepatic insulin signaling led to the decreased expression of *Pla2* genes, including *Pla2g4b*, *Pla2g16*, *Pnpla6* and *Pnpla4* without affecting *Lclat1* expression (Figure 6H,I). Moreover, DKO mice had lower LCL levels, specifically the 18:2–18:2–18:2 LCL species (Figure 6J,K), suggesting impaired generation of LCL from CL in the liver of DKO mice. These results demonstrate that disruption of hepatic insulin signaling dysregulates CL and LCL profiles in the liver.

### 3.7 Hepatic TGF‐β1 or Foxo1 Deficiency Attenuates Phospholipid Alternations in the Liver of DKO Mice

**FIGURE 7:**
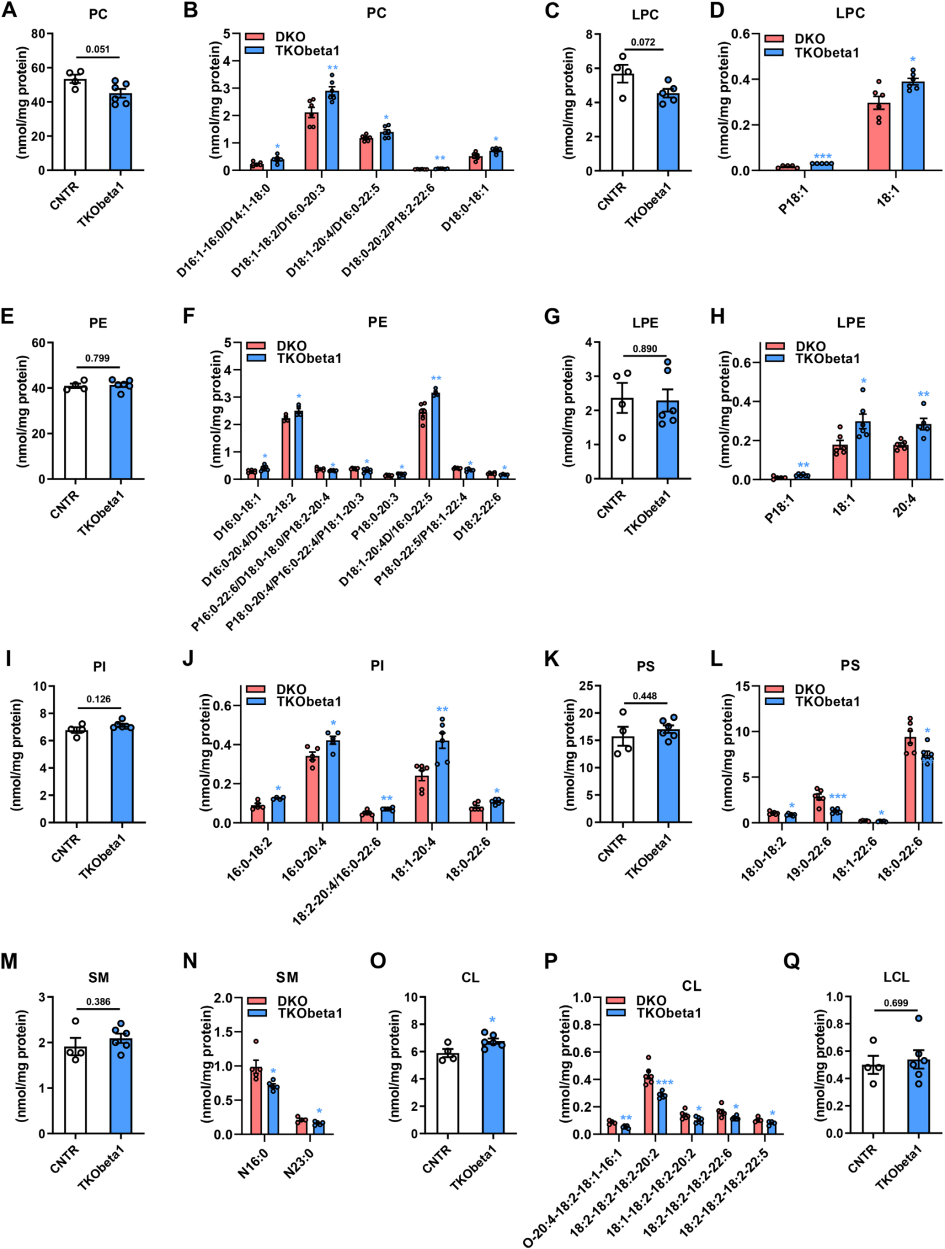
Hepatic TGF‐β1 or Foxo1 deficiency attenuates phospholipid alterations in the liver of DKO mice. (A) Total PC levels in the liver of TKObeta1 and CNTR3 mice. (B) Levels of hepatic PC molecular species in TKObeta1 and DKO mice. (C) Total LPC levels in the liver of TKObeta1 and CNTR3 mice. (D) Levels of hepatic LPC molecular species in TKObeta1 and DKO mice. (E) Total PE levels in the liver of TKObeta1 and CNTR3 mice. (F) Levels of hepatic PE molecular species in TKObeta1 and DKO mice. (G) Total LPE levels in the liver of TKObeta1 and CNTR3 mice. (H) Levels of hepatic LPE molecular species in TKObeta1 and DKO mice. (I) Total PI levels in the liver of TKObeta1 and CNTR3 mice. (J) Levels of hepatic PI molecular species in TKObeta1 and DKO mice. (K) Total PS levels in the liver of TKObeta1 and CNTR3 mice. (L) Levels of hepatic PS molecular species in TKObeta1 and DKO mice. (M) Total SM levels in the liver of TKObeta1 and CNTR3 mice. (N) Levels of hepatic SM molecular species in TKObeta1 and DKO mice. (O) Total CL levels in the liver of TKObeta1 and CNTR3 mice. (P) Levels of hepatic CL molecular species in TKObeta1 and DKO mice. (Q) Total LCL levels in the liver of TKObeta1 and CNTR3 mice. Data are presented as the means ± SEM. *p < 0.05, **p < 0.01, **p < 0.001, ****p < 0.0001 vs. CNTR or DKO group using *t*‐test.

Our recent study has shown that hepatic TGF‐β1 mediates the effect of hepatic signaling disruption on glucose and energy metabolism [11, 26]. To examine whether hepatic TGF‐β1 also contributes to insulin signaling disruption‐induced phospholipid alterations, we generated liver specific *Irs1*, *Irs2*, and *Tgfb1* triple knockout mice (TKObeta1) and compared the phospholipid profiles of these mice with DKO mice. Hepatic TGF‐β1 deficiency attenuated insulin signaling disruption‐induced decrease of total PC levels (TKObeta1 vs. CNTR3: 15% decrease, *p* = 0.051; DKO vs. CNTR: 20% decrease, *p* = 0.0029) (Figures 1A and 7A). Several PC species, including D16:1–16:0/D14:1–18:0; D18:1–18:2/D16:0–20:3; D18:1–20:4/D16:0–22:5; D18:0–20:2/D18:2–22:6; D18:0–18:1 were significantly increased in TKObeta1 mice compared to those of DKO mice (Figure 7B). Hepatic TGF‐β1 deficiency also ameliorated insulin signaling disruption‐induced decrease of total LPC levels (TKObeta1 vs. CNTR3: 20% decreases, *p* = 0.071; DKO vs. CNTR: 31% decrease, *p* = 0.0013) (Figures 1G and 7C). The alterations of P18:1; 18:1 LPC species in the liver of DKO mice were diminished by hepatic *Tgfb1* deletion (Figure 7D). While having similar total PE levels in the liver as control of mice, TKObeta1 mice displayed attenuated alterations of several PE species compared to those of DKO mice (Figure 7E,F). Moreover, hepatic TGF‐β1 deficiency blocked insulin signaling disruption‐induced reduction of total LPE levels (TKObeta1 vs. CNTR3: 4% decreases, *p* = 0.89; DKO vs. CNTR: 22% decrease, *p* = 0.028) (Figures 2J and 7G). Specifically, the decreases of P18:1; 18:1; 20:4 LPE species in the liver of DKO mice were diminished by hepatic *Tgfb1* deletion (Figure 7H). Furthermore, hepatic TGF‐β1 deficiency restored total PI, PS and SM levels, as well as several PI, PS and SM species in the liver in DKO mice (Figure 7I–N). TKObeta1 mice showed attenuated increases of total hepatic CL levels compared to DKO mice (TKObeta1 vs. CNTR3: 14.8% increase, *p* = 0.033; DKO vs. CNTR: 27.2% increase, *p* = 0.0024) (Figures 6A and 7O). Specifically, hepatic TGF‐β1 deficiency diminished insulin signaling disruption‐induced increases of several CL species, including O20:4–18:2–18:1–16:1, 18:2–18:2–18:2–20:2,18:1–18:2–18:2–20:2, 18:2–18:2–18:2–22:6, and 18:2–18:2–18:2–22:5 (Figure 7P). Hepatic TGF‐β1 deficiency blocked insulin signaling disruption‐induced decreases of hepatic LCL levels and increases of hepatic PG levels (Figure 7Q) Figure S1A,B. These results suggest that TGF‐β1 plays a critical role in mediating hepatic insulin signaling disruption‐induced alterations of phospholipids.

Foxo1, a key component of insulin signaling, mediates insulin signaling disruption‐induced metabolic alterations in several tissues, including liver, muscle, adipose tissue, and heart [27, 28]. We next generated *Irs1*, *Irs2*, and *Foxo1* triple knockout mice (TKOfoxo1) and found that hepatic *Foxo1* deletion significantly increased PC and LCL levels, while decreasing SM, CL, and ceramide levels in the liver of DKO mice (Figure S2A–S2D), suggesting that Foxo1 is required for insulin signaling disruption‐induced phospholipid (especially, PC, SM, CL, and LCL) alterations.

### 3.8 Hepatic TGF‐β1 or Foxo1 Deficiency Ameliorates the Alterations of Genes Related to Phospholipids Metabolism in the Liver DKO Mice

**FIGURE 8:**
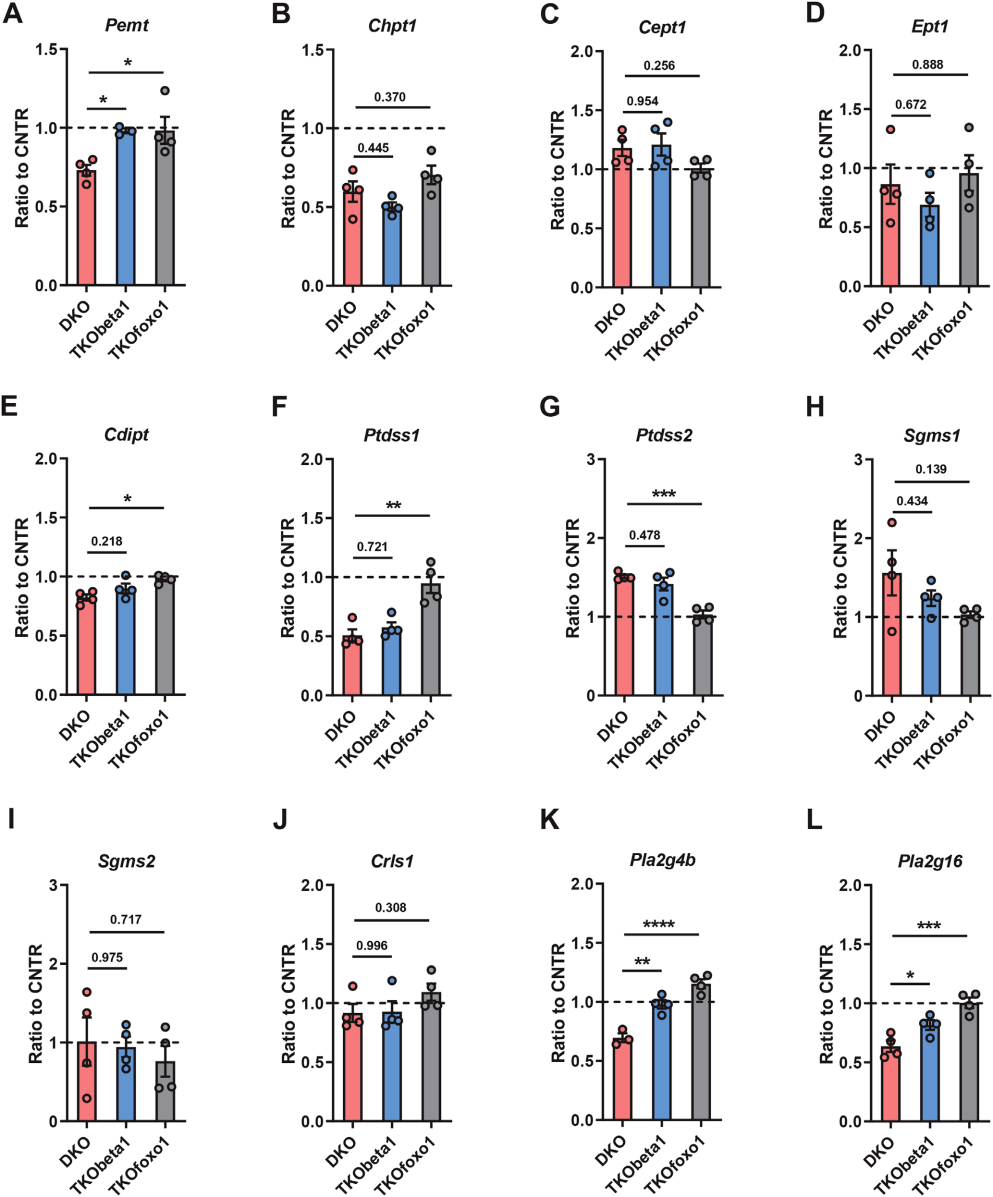
Hepatic TGF‐β1 or Foxo1 deficiency ameliorates the alterations of genes related to phospholipids metabolism in the liver of DKO mice. (A‐L) Relative expression (Ratio to CNTR levels) of *Pemt* (A), *Chpt1* (B), *Cept1* (C), *Ept1* (D), *Cdipt* (E), *Ptdss1* (F), *Ptdss2* (G), *Sgms1* (H), *Sgms2* (I), *Crls1* (J), *Pla2g4b* (K), and *Pla2g16* (L) in the liver of DKO, TKObeta1, and TKOfoxo1 mice. Data are presented as the means ± SEM. *p < 0.05, **p < 0.01, **p < 0.001, ****p < 0.0001 between assigned groups one‐way ANOVA tests followed by Tukey's multiple comparisons tests.

Our results demonstrated that disruption of hepatic insulin signaling modulated phospholipid profiles largely via regulating the expression of genes encoding key enzymes involved in phospholipid metabolism. Given that both hepatic TGF‐β1 and Foxo1 are key contributors to phospholipid alterations in DKO mice (Figure 7 and S2), we next examined whether hepatic *Tgfb1* or *Foxo1* deletion affected the expression of those genes. Compared to DKO mice, TKObeta1 and TKOfoxo1 mice showed a recovery of *Pemt* expression but no significant change in *Chpt1* expression in the liver (Figure 8A,B). Consistent with the observation that TKObeta1 and TKOfoxo1 mice had similar PE levels as DKO mice, hepatic *Tgfb1* or *Foxo1* deletion did not significantly affect *Cept1* and *Ept1* expression (Figure 8C,D). While TKObeta1 mice showed similar hepatic *Cdipt* expression to DKO mice, TKOfoxo1 mice showed restored expression of *Cdipt* in the liver of DKO mice (Figure 8E). Similarly, while hepatic *Tgfb1* deletion did not affect *Ptdss1* and *Ptdss2* expression, hepatic *Foxo1* deletion restored the expression of *Ptdss1* and *Ptdss2* in the liver of DKO mice (Figure 8F,G). Neither hepatic *Tgfb1* deletion nor *Foxo1* deletion affected *Sgms1*, *Sgms2*, and *Crls1* expression, suggesting that TGF‐β1 or Foxo1 promotes SM and CL levels independent of regulating these genes' expression (Figure 8H–8J). Interestingly, hepatic *Tgfb1* or *Foxo1* deletion significantly diminished the decrease of *Pla2g4b* and *Pla2g16* expression in DKO mice (Figure 8K,L), which potentially restored the lyso‐phospholipids (LPC, LPE, and LCL) levels in TKObeta1 and TKOfoxo1 mice (Figure 7C,G,Q and Figure S2D).

## 4 Discussion

In this study, we highlighted the importance of insulin signaling in the control of phospholipid metabolism. Our results include three important findings: (1) disruption of hepatic insulin signaling (DKO) altered phospholipid profiles and caused abnormal expression of genes related to phospholipid metabolism. (2) Hepatic TGF‐β1 or Foxo1 deficiency attenuated the alterations of phospholipids in the liver of DKO mice. (3) Hepatic TGF‐β1 or Foxo1 deficiency recovered the expression of genes related to phospholipid metabolism in DKO mice. Our study suggests that hepatic insulin signaling maintains phospholipids balance in the liver via TGF‐β1 or Foxo1.

Phospholipids are synthesized via two main pathways: *de novo* synthesis or converting from other phospholipids [29]. In this study, we demonstrated that hepatic insulin signaling disruption significantly altered the expression of genes that control these two enzymatic pathways, at least partially, via TGF‐β1 or Foxo1. In DKO mice, genes involved in PC synthesis, including *Pemt* and *Chpt1* were downregulated (Figure 1F). Further deletion of TGF‐β1 or Foxo1 restored the expression of *Pemt* but not *Chpt1*, suggesting that hepatic TGF‐β1 or Foxo1 deficiency rescued hepatic PC levels via promoting the conversion from PE to PC in DKO mice (Figures 7A, 8A and S2A). Previously, Laura et al. demonstrated that transcription factor Sp1 negatively regulates the expression of *Pemt* in the liver and adipocytes [30]. Foxo1 or Smad3, which are TGF‐β1 downstream mediators, coordinates with Sp1 in control target genes expression [31]. In DKO mice, the activation of TGF‐β1 or Foxo1 could potentially promote Foxo1 or Smad3 to form complexes with Sp1 to suppress *Pemt* expression. Additionally, we found that *Cdipt*, which controls PI synthesis, also decreased in DKO mice. Such alteration was diminished by further deletion of Foxo1 but not by TGF‐β1 deletion (Figure 8E). The improvement of PI synthesis in TKObeta1 mice is likely due to an increase of inter‐conversion using PA (Figure S1D,E) instead of *de novo* synthesis. In PS synthesis, *Ptdss1* was decreased while *Ptdss2* was increased in DKO mice, suggesting that the increased PS levels observed in DKO mice resulted from elevated conversion from PE to PS which is controlled by *Ptdss2* (Figure 4E,F). Interestingly, the expressions of *Ptdss1* and *Ptdss2* were restored to normal levels by further deletion of Foxo1, indicating that Foxo1 is a potential regulator of *Ptdss1* and *Ptdss2* expression. Unexpectedly, hepatic TGF‐β1 deletion blocked the hepatic insulin signaling disruption‐induced increase of PS without affecting the expressions of *Ptdss1* and *Ptdss2* in the liver of DKO mice. Previously, TGF‐β1 signaling has been shown to promote *de novo* serine synthesis [32, 33], which potentially contribute to the increase of hepatic serine levels observed in DKO mice (Figure 4G). Hepatic TGF‐β1 deletion might suppress PS synthesis in DKO mice via reducing substrate serine. In SM and CL synthesis, hepatic insulin signaling disruption barely affected the expression of key enzymes. The increased SM and CL levels in DKO mice were likely due to the elevated substrates levels for SM and CL synthesis, including ceramide and PG levels. Such alterations were attenuated by hepatic TGF‐β1 or Foxo1 deficiency, suggesting that hepatic insulin signaling disruption promotes the conversion of ceramide to SM and PG to CL via TGF‐β1 and Foxo1. Indeed, Foxo1 was previously reported to promote ceramide levels via Krüppel‐like factor‐5 (KLF5) [34]. TGF‐β1 signaling has been shown to cause cellular calcium accumulation, which can promote PG levels [35]. These findings support that hepatic TGF‐β1 and Foxo1 mediate the impacts of hepatic insulin signaling disruption on phospholipid synthesis.

In addition to phospholipid synthesis, the fatty acyl chain composition of phospholipids is also important to determine the properties of biological membranes and thereby affect membrane‐associated cellular processes [36]. The composition of fatty acyl chains in phospholipids is regulated by a remodeling process called Lands' cycle: upon phospholipids synthesis, a series of diacylation and reacylation reactions controlled by PLA2s and lysophopholipid acyltransferase (LPLAT), respectively, incorporate diverse fatty acids to form new phospholipid species [37]. In the present study, we demonstrated that hepatic insulin signaling disruption could cause phospholipids remodeling. The LPLATs involved in PC remodeling are lysophophatidylcholine acyltransferase (LPCATs), including LPCAT1, LPCAT2, LPCAT3 and LPCAT4 [36]. Here, we found that hepatic insulin signaling disruption significantly led to the increased expression of *Lpcat1* gene (Figure S1J–M). LPCAT1 prefers 16:0‐acyl‐CoA as an acyl donor to synthesize dipalmitoyl PC (32:0) [38, 39]. Indeed, we found that dipalmitoyl PC (32:0) species was increased though the majority of PC species were decreased in DKO mice (Figure 1D). Hepatic TGF‐β1 or Foxo1 deficiency restored the expression of *Lpcat1* gene (Figure S1J), and hepatic TGF‐β1 deletion blocked hepatic insulin signaling disruption‐induced increase of dipalmitoyl PC (32:0) species in the liver. Moreover, hepatic LPC was downregulated in DKO mice, along with impaired expression of PLA2s, including *Pla2g4b*, *Pla2g16*, *Pnpla6* and *Pnpla7* (Figure 6H). Hepatic TGF‐β1 or Foxo1 deficiency restored the expression of *Pla2g4b* and *Pla2g16* gene (Figure 8K,L), and hepatic TGF‐β1 deletion diminished hepatic insulin signaling disruption‐induced decreases of total LPC and P18:1, P18:0, and 18:1 LPC species (Figure 7C,D). These results highlighted the crucial role of insulin signaling in control of PC remodeling via TGF‐β1 or Foxo1. Furthermore, the fatty acyl chain composition of PE was largely altered in the liver of DKO mice (Figure 2C). Especially, the most abundant PE species (D38:6) was elevated in DKO mice though total PE levels were unaffected. Similarly, hepatic PE, PS, SM and CL profiles were also remodeled in DKO mice: PS species containing 18:2–18:2, 18:1–20:4 and 18:0–20:4 fatty acyl chains, SM species containing 22:1, 22:0 and 23:1 fatty acyl chains, and CL species containing 18:2–18:2–18:2–18:1 fatty acyl chains were significantly reduced despite that total PS, SM and CL levels were increased (Figure 4C,D). Such hepatic insulin signaling disruption‐induced phospholipid remodeling was blocked or attenuated in TKObeta1 mice, suggesting that hepatic TGF‐β1 plays an important role in phospholipid remodeling, especially under insulin resistance conditions.

Phospholipids are important for maintaining cellular and organelles' membrane structure and functions, while alterations of phospholipids in the liver are associated with the pathogenesis of liver diseases such as NAFLD and NASH [2]. In the progression of NAFLD and NASH, both PC and PE contents are reduced in the liver [40, 41]. Decreased PC in NAFLD contributes to impaired very low‐density lipoproteins (VLDL) secretion, promoting hepatic steatosis [42, 43]. In this study, we demonstrated a decrease of PC in DKO mice, due to impaired PC synthesis from both CDP‐choline pathway and PEMT pathway (Figure 1F). Consequently, VLDL secretion was dramatically impaired in DKO mice [44]. Impaired PE synthesis provides excess substrate for TG production, causing excess hepatic lipid accumulation in NAFLD patients [45]. In addition, the PC/PE ratio was also reduced in the liver of patients with NASH, facilitating hepatocyte damage and hepatic inflammation [40, 46, 47]. Although total PE levels were not altered, hepatic PC/PE ratio was significantly reduced in DKO mice (Figure 2I), which may contribute to the exacerbated liver injury in hepatic insulin resistant mice [48]. Alterations in mitochondria‐enriched phospholipids, such as CL and PG have also been observed in the liver of NAFLD/NASH patients [2]. In DKO mice, we found that hepatic CL levels were increased as a result of elevated PG levels (Figure 6A,G). A recent study demonstrated the CL is required for mitochondrial fusion [49]. Indeed, increased mitochondrial fusion were observed in the liver of DKO mice [50]. Moreover, reduced hepatic PI levels were reported in NASH patients [40]. While dietary PI supplementation prevents the development of NAFLD in rats [51], disturbances of PI synthesis promotes NASH development [52], suggesting a protective role of PI in NASH progression. Here, we found that hepatic PI levels were also decreased in DKO mice. These similar alterations of phospholipids (including PC, PC/PE, CL, PG, and PI) observed in DKO mice and NAFLD patients are consistent with the fact that hepatic insulin resistance is highly associated with NAFLD development [53]. The alterations of phospholipids potentially caused hepatic dysfunction in insulin resistance conditions [48, 54]. Our recent studies demonstrated that reciprocal regulation of hepatic TGF‐β1 and Foxo1 promotes NAFLD, liver fibrosis and injury [11, 55]. The improvement of phospholipid metabolism by hepatic TGF‐β1 or Foxo1 deletion may contribute to the restored liver function observed in TKObeta1 and TKOfoxo1 mice [11, 44, 50]. Hepatic TGF‐β1‐ or Foxo1‐controlled phospholipid synthesis and remodeling may bring novel insights in our understanding of insulin signaling in control of liver function.

## Author Contributions

Quan Pan designed the study, carried out the research, interpreted the results, and wrote the manuscript; Meixia Pan performed research and reviewed the manuscript; Weiqi Ai, Wanbao Yang, Wen Jiang, and Xianlin Han reviewed the manuscript; Shaodong Guo designed the study, analyzed the data, reviewed, and revised the manuscript.

## Funding

This work was supported by Foundation from the National Institutes of Health (NIH): (R01DK095118), (R01DK120968), and (R01DK124588). American Diabetes Association (ADA): (1 ‐ 15 ‐ CD ‐09). Texas A&M University faculty start‐up funds. U.S. Department of Agriculture (USDA): Hatch (1010958).

## Disclosure

The authors have nothing to report.

## Conflicts of Interest

The authors declare no conflicts of interest.

## Supporting information


**Figure S1:** The effect of hepatic TGF‐β1 deficiency on hepatic PG and PA levels, and on hepatic genes expression in DKO mice.
**Figure S2:** The effect of hepatic Foxo1 deficiency on hepatic PC, SM, CL, LCL, and ceramide levels in DKO mice.

## Data Availability

No new data.
