# Supplementary material for: Disruption of Hepatic Insulin Signaling Causes Phospholipid Dysregulation in Mice
Source: FASEB J. 2026 Feb 24;40(4):e71613. doi: 10.1096/fj.202504306R (PMC12931579; doi:10.1096/fj.202504306R)
Supplement: Supplementary file 1 — Figure S1: The effect of hepatic TGF‐β1 deficiency on hepatic PG and PA levels, and on hepatic genes expression in DKO mice. Figure S2: The effect of hepatic Foxo1 deficiency on hepatic PC, SM, CL, LCL, and ceramide levels in DKO mice. [file FSB2-40-e71613-s001.pdf]

Figure S1

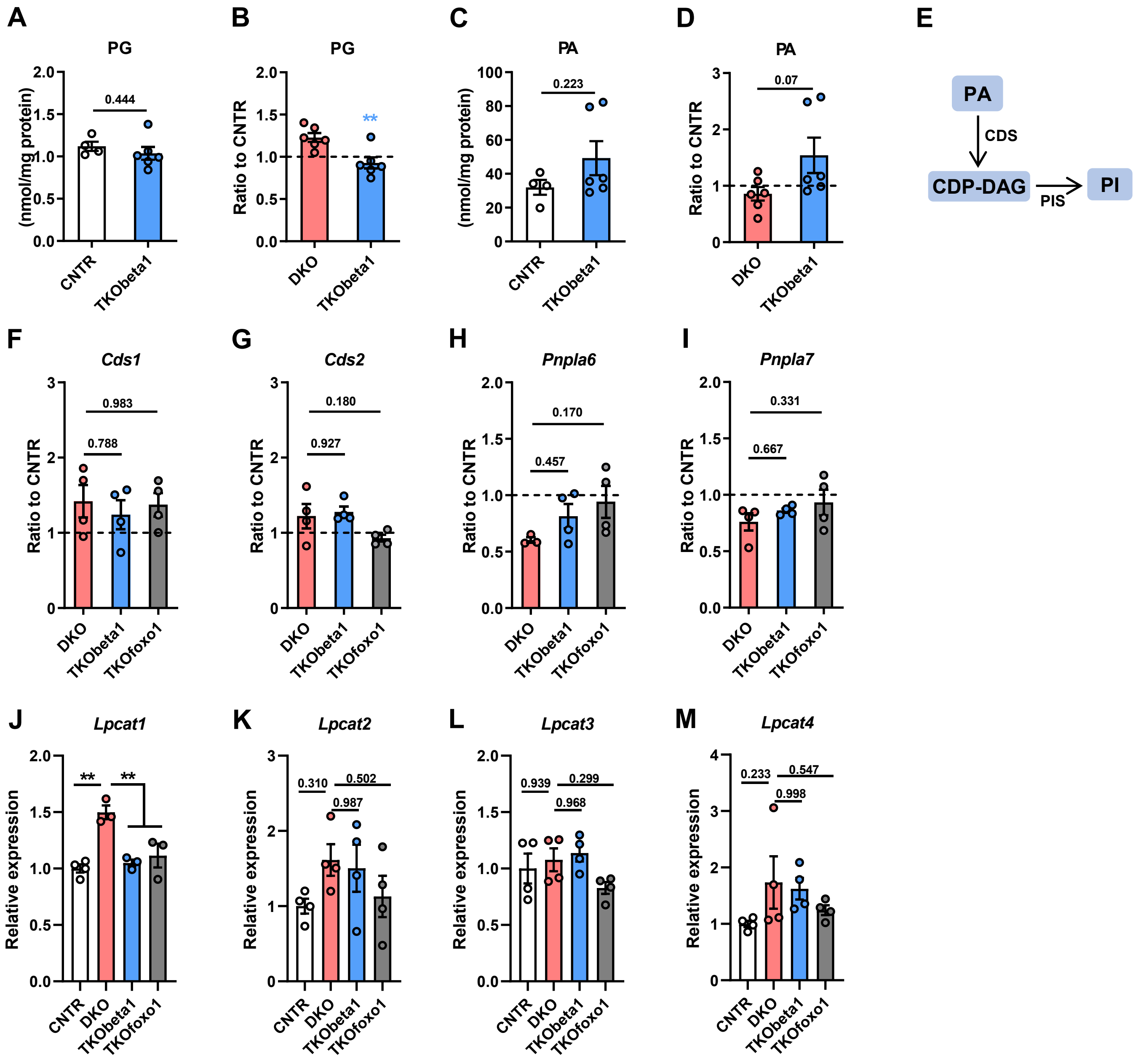

Figure S1. The effect of hepatic TGF-β1 deficiency on hepatic PG and PA levels, and on hepatic genes expression in DKO mice. **(A)** Total PG levels in the liver of TKObeta1 and CNTR mice. **(B)** Relative PG levels in the liver of DKO and TKObeta1 mice (Ratio to CNTR mice). **(C)** Total PA levels in the liver of TKObeta1 and CNTR mice. **(D)** Relative PA levels in the liver of DKO and TKObeta1 mice (Ratio to CNTR mice). **(E)** Diagram showing the synthesis of PI from CDP-DAG by PI synthase (PIS) and the synthesis of CDP-DAG from PA by CDP-Diacylglycerol Synthases (CDS). **(F-I)** Relative expression (Ratio to CNTR levels) of *Cds1* **(F)**, *Cds2* **(G)**, *Pnpla6* **(H)** and *Pnpla7* **(I)** in the liver of DKO, TKObeta1, and TKOfoxo1 mice. **(J-M)** Relative expression of *Lpcat1* **(J)**, *Lpcat2* **(K)**, *Lpcat3* **(L)** and *Lpcat4* **(M)** in the liver of CNTR, DKO, TKObeta1, and TKOfoxo1 mice. Data are presented as the means ± SEM. ** p< 0.01 vs DKO or between assigned groups using one-way ANOVA or t-test.

Figure S2

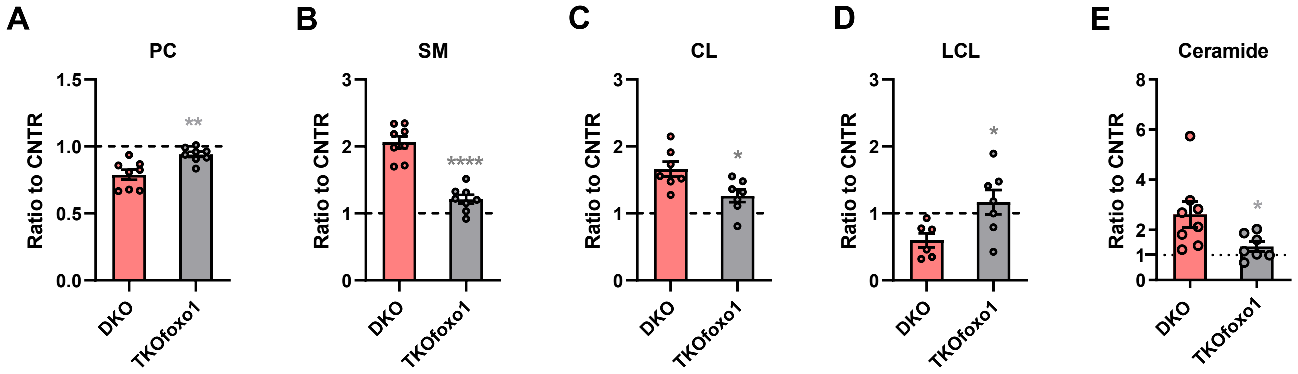

Figure S2. The effect of hepatic Foxo1 deficiency on hepatic PC, SM, CL, LCL, and ceramide levels in DKO mice. **(A-E)** Relative PC **(A)**, SM **(B)**, CL **(C)**, LCL **(D)**, ceramide **(E)** levels in the liver of DKO and TKOfoxo1 mice (Ratio to CNTR mice). Data are presented as the means ± SEM * p< 0.05, ** p< 0.01, **** p< 0.0001 vs CNTR or DKO group using t-test. .
